# Double Barreled Wet Colostomy: Initial Experience and Literature Review

**DOI:** 10.1155/2014/961409

**Published:** 2014-12-03

**Authors:** Luis Salgado-Cruz, Eloy Espin-Basany, Francesc Vallribera-Valls, Jose Sanchez-Garcia, Luis Miguel Jimenez-Gomez, Marc Marti-Gallostra, Ana Garza-Maldonado

**Affiliations:** ^1^Colorectal Surgery Unit, Vall d'Hebron University Hospital, Universitat Autonoma de Barcelona, Barcelona, Spain; ^2^Instituto de Cirugía Centro Médico Zambrano Hellion-Tec Salud, Batallón de San Patricio 112, Real de San Agustín, 66278 San Pedro Garza García, NL, Mexico; ^3^School of Medicine and Health Sciences, Tecnológico de Monterrey, Mexico

## Abstract

*Background*. Pelvic exenteration and multivisceral resection in colorectal have been described as a curative and palliative intervention. Urinary tract reconstruction in a pelvic exenteration is achieved in most cases with an ileal conduit of Bricker, although different urinary reservoirs have been described.* Methods*. A retrospective and observational study of six patients who underwent a pelvic exenteration and urinary tract reconstruction with a double barreled wet colostomy (DBWC) was done, describing the preoperative diagnosis, the indication for the pelvic exenteration, the complications associated with the procedure, and the followup in a period of 5 years. A literature review of the case series reported of the technique was performed.* Results*. Six patients had a urinary tract reconstruction with the DBWC technique, 5 male patients and one female patient. Age range was from 20 to 77 years, with a medium age 53.6 years. The most frequent complication presented was a pelvic abscess in 3 patients (42.85%); all complications could be resolved with a conservative treatment.* Conclusion*. In the group of our patients with pelvic exenteration and urinary tract reconstruction with a DBWC, it is a safe procedure and well tolerated by the patients, and most of the complications can be resolved with conservative treatment.

## 1. Introduction

Pelvic exenteration has been described since the 1940s as both a curative and palliative intervention in gynecologic cancer [1, 2]. During the mid 1940s, abdominoperineal resection, radical cystectomy, and radical hysterectomy were established as a treatment option for neoplastic disease involving these organs. Due to its great morbidity, the combination of such procedures was considered as almost prohibitive.

Alexander Brunschwig in 1948 described the first pelvic exenterations in advanced carcinoma of the cervix [2, 3]. Appleby in 1950 described the proctocystectomy in 8 patients with rectal carcinoma, and so Brintall and Folcks described the “pelvic viscerectomy” [1].

Historically, the morbidity and mortality of the pelvic exenteration have been 50% and 10%, respectively [4]. Multivisceral resection in colorectal carcinoma increases survival and can be performed with curative or palliative intentions [4, 5].

One of the problems that surgeons face during a pelvic exenteration is the reconstruction of the urinary tract. Brunschwig [6] and later Daniel reported the first cases of an ureterocolostomy, after a pelvic exenteration in 1948. The urinary complications and the difficulties with the stoma output forced to create new technical alternatives, reason why this technique was abandoned.

In 1940, Bricker described the use of an ileal conduit to replace the urinary bladder in the Ellis Fischel Cancer Hospital [7]. Since then, the ileal conduit has been the standard procedure for urinary bladder reconstruction in pelvic exenteration [3].

The patient comorbidities, associated with the extent of the pelvic exenteration, make this procedure to be prone to more complications in patients on which a Bricker ileal conduit and a stoma from fecal stream are obviously necessary. The altered body image of the patients, who have a stoma, has forced to search for new alternatives [8]. In 1989, Carter redesigned the wet colostomy technique, to avoid the complications linked with the original technique described by Brunschwig. Carter described the technique of the double barreled wet colostomy (DBWC) in a patient with a loop sigmoid colostomy and an actinic colovesical fistula and the patient refused to have an additional stoma [9].

The aim of this study is to make a description of the first patients treated with a DBWC in the Colorectal Unit in the Vall d'Hebron University Hospital and the literature review of this surgical technique.

## 2. Methods

Observational and retrospective study of the patients with a pelvic exenteration and urinary tract reconstruction with a double barreled wet colostomy, between May 2006 and October 2010, was done. We registered the clinical data of the patients, demographic data, clinical characteristics of the patients, and surgical indication of the procedure. The early and late morbidity were recorded, as postoperative followup. Absolute and relative frequencies of every data were recorded. The literature review of the DBWC was performed, and a comparison of the results with this group of patients was done.

### 2.1. Surgical Technique

**Figure 1:**
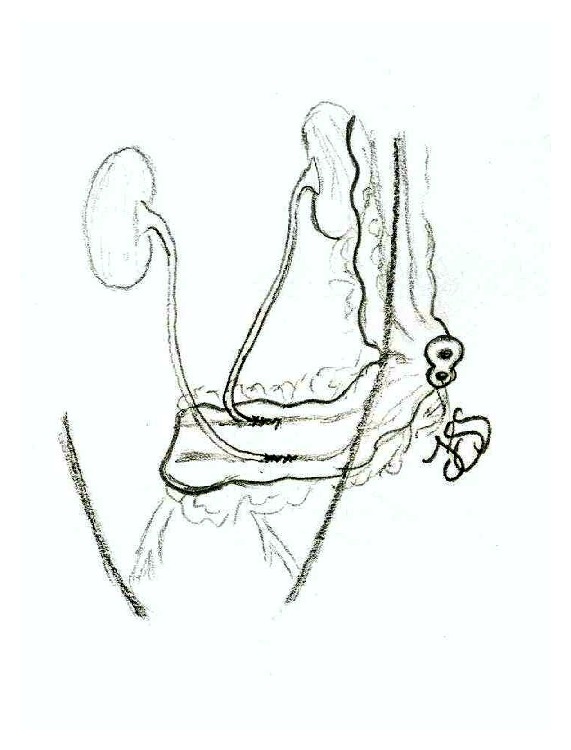
Schematic representation of double barreled wet colostomy.

In all patients, and once the pelvic exenteration was performed, both urethers are freed from the retroperitoneum with care to protect their blood supply. The last 15 to 20 cm of colon is used as urinary reservoir. Muscular and submuscular dissection are made in the taenia, and an antireflux ureterocolic anastomosis is made; ureters are sutured with single J catheters placed. A loop colostomy is constructed with eversion of the fecal stream. Finally, the urinary reservoir is fixed to the retroperitoneum in order to avoid pulling from the ureterocolonic sutures (Figure 1). The urethral catheters are left in place for three weeks and removed previous radiologic assessment that confirms that no complications are present in the ureterocolonic anastomosis.

## 3. Results

A total of 6 double barreled wet colostomies have been made, six male patients and one female patient. Age range was from 20 to 77 years, with a medium age 53.6 years. Two of the patients were operated with a diagnosis of rectal adenocarcinoma, two patients with sigmoid colon adenocarcinoma, one patient with a neuroendocrine prostate tumor, and a patient with local recurrence of rectal adenocarcinoma, who previously had an abdominoperineal resection (APR) with in block hysterectomy 5 years prior to this intervention. These patients had adjuvant radiotherapy.

The clinical stages for colorectal adenocarcinoma were one patient IIIB, one IIB, two stages IV, and one stage IIIC. The indication for pelvic exenteration was for locally advanced disease which infiltrated the urinary bladder and/or other bowel segments, a local recurrence, and, in one of the stage IV patients, a peritoneal implant was found in the pelvis. The other stage IV patient had single hepatic metastatic lesions. Two of patients operated already had a colostomy, a sigmoid loop colostomy in one of the stage IV patients, and a terminal colostomy in the patient with a previous APR (Table 1). Pelvic exenteration was completed in 4 of the patients with a low Hartmann procedure, and, in one patient, an intersphincteric anal resection was performed. In the patient with the local recurrence, the cystectomy was performed associated with an in block intestinal resection. In one of the stage IV patients, beside the pelvic exenteration, an ileocecal resection, a resection of a peritoneal implant in the right iliac fossa, had to be performed. All interventions except for the patient with the liver metastasis were considered R0. One of the patients had a synchronic prostate cancer as an incidental finding.

In the immediate postoperative followup (<30 postoperative days), two of the patients presented ileus; three patients developed a pelvic abscess resolved with a transuretral drainage with a Foley catheter and intravenous antibiotic. Two patients had central venous access sepsis. One patient had a postoperative bleeding from a branch of the left hypogastric artery in the first postoperative hours and was treated with interventional radiographic embolization. The patient with intersphincteric anal resection required VAC therapy to control a perineal wound infection. No patient had a urinary tract related complication in the early postoperative period (<30 days) (Table 1).

An average followup of 19.5 months was achieved, with a range of 7 to 60 months. Two of the patients had disease recurrence. A retrogastric implant was diagnosed 11 months after surgery in the patient with the prostate neuroendocrine tumor. The patient with a peritoneal implant in the right iliac fossa had a peritoneal recurrence in the 5th postoperative month. A patient had a stomal prolapse in the third year of followup, and same patient developed a complicated perineal sinus posterior to a pelvic abscess associated with low Hartmann's procedure. For this patient, a perineal resection of anus and fistulous tracts with a VRAM (Vertical Rectus Abdominal Muscle) flap reconstruction was done.

Only one patient has developed a septic urinary complication, in the late postoperative period. This complication was associated with the presence of a ureteral occlusion secondary to peritoneal carcinomatosis in the patient with a prostate neuroendocrine tumor, treated with a percutaneous nephrostomy. To date, only a metabolic complication has been registered consistent with mild hypokalemia, treated with oral potassium. No complications related to the ureterocolonic anastomosis have been found (Table 1).

Of the 6 patients during followup, 4 are alive. Two of the deaths were due to disease progression. The patient with hepatic metastasis has a disease progression with lesions in both lungs (Table 1).

## 4. Discussion

Anterior resection of the rectum, radical hysterectomy, and radical cystectomy were rarely simultaneous surgical interventions in the early years of 20th century, because they were associated with high morbidity and mortality. The evolution of perioperative care has allowed the surgeons to perform more radical procedures with curative or palliative intention. Once pelvic exenteration was established, surgeons faced the difficulty of urinary transit reconstruction. Brunschwig was the first surgeon who performed the wet colostomy technique, a procedure adopted by other surgeons in the further years [2, 3]. The metabolic and septic complications, as the difficulties in the stomal management, discouraged other surgeons to use this technique, searching for new alternatives for urinary tract reconstruction. Bricker described the ileal conduit technique as a safe and easier approach for urinary tract reconstruction than the wet colostomy proposed by Brunschwig [7].

One of the problems that has led to stake the wet colostomy technique was the denial of a patient that already had an intestinal stoma to have an additional urinary stoma. Carter in 1989 modified the technique of the wet colostomy originally described by Brunschwig and created the double barrel wet colostomy technique that avoids mixing the urinary flow with fecal stream. With this technique, Carter achieved lower urinary tract infection rates and an easier way to manage the stomal output, changing liquid feces from the old technique to semiformed feces, continuous urinary output, and better acceptance from the patients having only one stoma [9].

Double barreled wet colostomy is now used in cases of pelvic exenteration due to gynecological, urological, and/or colorectal primary or recurrent malignancies [2, 3, 10–16] (Table 2).

In the group of patients analyzed in this series, almost all but one patient were operated for colorectal adenocarcinomas, just one with a prostate neuroendocrine tumor. We have found that it is a safe procedure even in patients 70 years old or older, as other authors that have performed the procedure in patients in the eight decade of life with good perioperative results [2, 13–15] (Table 1).

Patients with previous stomas because of bowel occlusion secondary to actinic complications or primary neoplasm are not excluded to be candidates to this urinary tract reconstruction [2, 10, 14]. Two of our patients already had an intestinal stoma, prior to this surgical procedure. In these cases, the original stoma is converted to a DBWC.

All of the patients in this series were operated because of malignant disease. In other published series, patients with benign disease were included (actinic complications and fistulous disease) (Table 2). Five were primary disease and only one patient has been operated for recurrent disease. Four patients were operated with curative intention. Two palliative procedures were performed, as in other series where palliative procedures were included (Table 5).

Different complications can be seen in the patients with urinary tract diversions. In the reviewed literature of patients with double barreled wet colostomy, septic complications may occur in the early postoperative period as abscesses and intestinal fistulas [8, 17] (Tables 3 and 4).

Complications associated with the ureterocolonic anastomoses may generate morbidity in the early and late postoperative periods. In the early period, urinary fistulas and urinary tract infections are more common, and, in the late postoperative period, the anastomoses stenosis, with the secondary hydronephrosis, is reported more frequently. Urinary stones have been reported in the colonic urinary reservoir. Other early complications with lower incidence are the ones associated with the perineal wound, postoperative ileus, and central venous access infection [2, 3, 10, 13–15]. Only Carter has reported the presence of a metabolic acidosis associated with the urinary reservoir, complication that can be seen in any patient with an intestinal urinary reservoir [8, 10, 17]. Another late complication reported in the DBWC is stomal complications [2, 13]. In our group of patients, the most frequent complication was the pelvic abscess, present in 3 patients, followed by postoperative ileus in 2 patients, and sepsis related to central line infection (2 patients). No complications in the ureterocolonic anastomosis have been encountered, probably because of the care taken when performing the ureter dissection, the antireflux anastomosis, and uretheral tutors in place for three weeks after the procedure. In late complications, a stomal prolapse and a persistent perineal sinus have been presented in the same patient, patient that needed surgical correction. Only one urinary tract infection associated with ureteral occlusion secondary to disease recurrence has been found, but it is important to mention that asymptomatic bacteriuria is reported by other authors and not searched in our group [12].

The perioperative mortality reported in the different published series of patients with a DBWC ranges from 0 to 11.56% [2, 3, 10–16]. No mortality in the perioperative period was found in our case series (Table 5). In our group of patients, we found only one complication related to the urinary reservoir (urinary tract infection). All of our patients presented a complication during the early (<30 days) or late (>30 days) postoperative period, although the complications had no repercussion on postoperative survival, and the majority were resolved in a conservative manner (Tables 1 and 5).

One of the problems that the patient with an intestinal urinary reservoir faces is an increased risk of carcinoma of intestinal origin in the ureterocolonic anastomosis [17, 18], a reason why this patient should be followed up in a long term. It is estimated that, in patients with a DBWC, the risk to develop a neoplasia can be as high as 7000 times the general population less than 25 years of age [18]. The mechanism can be related to the production of nitrosamines from the bacteria present in the urinary reservoir [17, 18]. No carcinomas related to the urinary reservoir have been encountered, although it is a short followup for this kind of complication.

To date, considering that no quality of life assessment has been performed, all our patients are satisfied with the procedure and deny having problems with stomal management. Lopes de Queiroz et al. [3], using the QLQ-C30 questionnaire, made a quality of life analysis, in 5 patients of the 9 who performed the DBWC, and reported high functional results and global improvement in their health status.

## 5. Conclusion

In our initial experience, we can conclude that the pelvic exenteration and urinary tract reconstruction with a DBWC are safe procedures and well tolerated by the patients. Although can be associated with certain morbidity, most of the complications could be resolved in a conservative manner.

A comparative of the different urinary reservoirs and the DBWC should be performed, where a quality of life assessment could be taken in every type of reservoir. A study of these characteristics will have to be a multicentric study, for the particularities of the patients needed.

For the risk of increase in neoplasia of the urinary reservoir, we suggest that the procedure should be used in patients committed to long term followup or with a not very long life expectancy.

## Figures and Tables

**Table 1:** Patients with DBWC at University Hospital Vall d'Hebron. UTI, urinary tract infection.

Patients who underwent DBWC at UHVH.						
Age/gender	Diagnosis	Previous stoma	Indications for surgery	Early complications (<30 days)	Late complications (>30 days)	Followup (months)
44/M	Rectal carcinoma stage IIIB	No	Infiltration of bladder trigone and prostate.	Paralytic ileus	Pelvic abscess, perineal sinus, stoma prolapse, and hypokalemia	Disease-free (60), VRAM reconstruction after perineal sinus resection.
20/M	Prostate neuroendocrine tumor	No	Infiltration of bladder trigone and rectum.	Hemorrhage, pelvic abscess	UTI	Disease recurrence (11). Died 13 months postoperatively.
77/M	Sigmoid carcinoma stage IV	No	Infiltration of bladder trigone, terminal ileum, and pelvic peritoneal implant.	Central catheter infection	None	Disease recurrence (5). Died 7 months postoperatively.
75/M	Sigmoid carcinoma stage IIB	No	Infiltration of bladder and prostate. Synchronous prostate carcinoma.	Pelvic abscess, paralytic ileus, central catheter infection	Actinic enteritis	Disease-free (16).
58/M	Rectal carcinoma stage IV	Yes	Infiltration of bladder	Wound infection	None	Disease progression w/lung metastasis (10).
48/F	Recurrent rectal carcinoma	Yes	Infiltration of bladder and intestinal loop	Wound infection	None	Disease-free (11)

**Table 2:** Diagnosis in DBWC patients. The literature review.

Diagnosis of patients with DBWC	
Diagnosis	*n*
Cervical carcinoma	58
Rectal carcinoma	42
Endometrial carcinoma	22
Actinic complications	17
Bladder carcinoma	9
Prostate carcinoma	7
Colon carcinoma	5
Vulvar carcinoma	4
Vaginal carcinoma	2
Ovarian carcinoma	2
Dehiscence urostomy [Bricker]	2
Anal carcinoma	1
Sacrococcygeal carcinoma	1
Pelvic carcinoma	1
Pelvic trauma	1
Hernia cystostomy	1
Neurogenic bladder and incontinence	1
Scrotal-urethra fistula	1
Rectovesical fistula	1
Primitive neuroectodermal tumor of the pelvis	1

**Table 3:** Reservoir related complications. The literature review.

Reservoir related complications	
Diagnosis	*n*
Hydronephrosis	3
Metabolic complications	1
Urinary tract infection	5
Urinary fistula	5
Torsion-necrosis	3
Stoma-related complications	3
Ureterocolonic stenosis	2
Lithiasis	1

**Table 4:** General complications in DBWC patients. The literature review.

General complications in the DBWC.	
Diagnosis	*n*
Intestinal fistula	4
Wound infection	4
Pelvic abscess	2
Paralytic ileus	7
Postoperative hemorrhage	2
Sepsis (central venous catheter)	2
Evisceration	3

**Table 5:** Published literature in DBWC. UTI, urinary tract infection. (Modified from Golda et al. [14] Dis Colon Rectum 2010; 53: 822–829).

Published literature in DBWC.											
Author	Year	*n*	Primary tumor (%)	Recurrence (%)	Palliative procedure (%)	Benign disease (%)	Perioperative Mortality (%)	Postoperative complications (%)	Reinterventions	UTI	Followup (months)
Carter et al. [10]	1994	11	6 (54.54)	3 (27.27)	—	2 (18.18)	1 (9.09)	6 (54.54)	0	0	1–80
Takada et al. [11]	1995	2	2 (100)	—	—	—	0	0	0	0	25–30
Osorio Gullón et al. [12]	1997	13	5 (38.46)	8 (61.53)	—	—	0	2 (15.38)	1 (7.69)	1 (7.69)	8–68
Blanco Díez et al. [16]	2003	7	1 (14.28)	2 (28.57)	—	4 (57.14)	0	0	0	0	NE
Guimaraes et al. [2]	2006	56	—	53 (94.64)	—	3 (5.35)	4 (7.14)	27 (48.21)	11 (19.64)	0	24
Lopes de Queiroz et al. [3]	2006	9	5 (55.55)	4 (44.44)	—	—	1 (11.11)	1 (11.11)	1 (11.11)	1 (11.11)	40
Kecmanovic et al. [15]	2008	38	24 (63.15)	—	9 (23.68)	5 (13.15)	0	6 (15.7)	0	0	48
Sukumar et al. [13]	2010	12	12 (100)	—	—	—	0	8 (66.66)	0	2 (16.66)	64
Golda et al. [14]	2010	41	23 (56.09)	7 (17.07)	1 (2.43)	11 (26.82)	1 (2.43)	27 (65.85)	10 (24.4)	4 (9.8)	1–156
**Present study**	**2011**	**6**	**5 (83.3)**	**1 (16.66)**	**2 (33.33)**	**0**	**0**	**6 (100)**	**1 (14.28)**	**1 (14.28)**	**7**–**61**
